# Increased Early Postoperative Complication Rate after Osteoporotic Hip Fracture in Patients with Low 25 (OH) Vitamin D Levels

**DOI:** 10.3390/nu16121917

**Published:** 2024-06-18

**Authors:** Andrea Fink, Paul Puchwein, Astrid Fahrleitner-Pammer, Michael Eder-Halbedl, Gerwin Alexander Bernhardt

**Affiliations:** 1 Department of Orthopaedics and Trauma, Medical University of Graz, 8036 Graz, Austria; andrea.fink@medunigraz.at; 2 Division of Endocrinology and Diabetology, Medical University of Graz, 8036 Graz, Austria; 3 Department of Orthopedics and Traumatology, LKH Feldbach-Fürstenfeld, Ottokar-Kernstock-Straße 18, 8330 Feldbach, Austria; 4 Orthochirurg, Kärntner Straße 1, 8770 St. Michael, Austria

**Keywords:** vitamin D, postoperative complication, hip fracture, operation, osteoporosis

## Abstract

This study investigated the association of preoperative 25-hydroxy (25 (OH)) vitamin D levels with postoperative complications in osteoporotic hip fracture patients following surgery. We hypothesized that patients with low concentrations of 25 (OH) vitamin D might have an increased risk of developing adverse outcomes. Between January 2019 and December 2020, a retrospective observational study was conducted, including low-energy fragility fractures at the proximal femur. Regarding preoperative 25 (OH) vitamin D levels, patients were divided into two groups (<30 ng/mL and ≥30 ng/mL). Early and late postoperative complications were assessed and graded according to the Clavien–Dindo classification system. Logistic regression analysis was performed to demonstrate the association between preoperative 25 (OH) vitamin D levels (<30 ng/mL, ≥30 ng/mL) and postoperative complications after adjusting for age and sex. Of 314 patients, 222 patients (70.7%) had a 25 (OH) vitamin D level of <30 ng/mL. The mean serum 25 (OH) vitamin D level was 22.6 ng/mL (SD 13.2). In 116 patients (36.9%), postoperative complications were observed, with the most occurring in the short term (95 patients, 30.2%). Late postoperative complications were present in 21 patients (6.7%), most graded as Clavien I (57.1%). Logistic regression analysis identified a low vitamin D level (<30 ng/mL) as an independent risk factor for early postoperative complications (OR 2.06, 95% CI 1.14–3.73, *p* = 0.016), while no significant correlation was found in late complications (OR 1.08, 95% CI 0.40–2.95, *p* = 0.879). In conclusion, preoperative 25 (OH) vitamin D serum level might be an independent predictor for early postoperative complications. However, future studies are warranted to determine risk factors for long-term complications and establish appropriate intervention strategies.

## 1. Introduction

Osteoporotic hip fractures typically occur after a low-level trauma equivalent to a fall from a standing height or less and are gradually becoming a major global concern [1]. The worldwide lifetime risk of osteoporotic fragility fractures of the hip, vertebral spine, and distal radius in women is 40% [2], and it is estimated that the worldwide incidence of hip fracture will double by 2050 [3]. The primary treatment strategy is surgery, focusing on effective reconstructive fixation techniques or joint replacement to provide solid stability and restore patients’ quality of life to its best. Even though satisfactory surgical outcomes are nowadays expected in most patients, osteoporotic hip fractures frequently predispose patients to further complications occurring after surgery. According to the Community-Based Hip Fracture Registry of Inacio et al., 22.1% of patients who were treated operatively after a hip fracture were readmitted to a hospital within 92 days due to postoperative complications [4]. Similarly, several other studies have pointed out the high incidence of complications arising directly after hip fracture surgery, either during hospitalization or after discharge [5,6,7], such as wound infection, dislocation, failure of fixation, peri-prosthetic fracture, need for revision surgery, etc. [6]. The latter leads to a detrimental burden of patients not fully recovering from surgery [8], as well as economic consequences due to prolonged hospitalization or readmission, increased use of medications and supportive devices, and higher probability of long-term care [9].

Various preoperative factors may influence a patient’s postoperative medical condition. The latest literature has highlighted the potential effect of 25-hydroxy (25 (OH)) vitamin D level on postoperative morbidity, considering vitamin deficiency as a significant risk factor for adverse outcomes [10,11,12]. As vitamin D plays a key role in the overall regulation of the cardiovascular, skeletal, endocrine, and immune systems [13], hypovitaminosis D may have potential implications for surgery and surgical outcomes as well. A systematic review found no consistency in the benefits of vitamin D supplementation in a number of highly heterogeneous studies [14]. Nevertheless, to date, no previous study has investigated postsurgical outcomes after osteoporotic hip fractures. By conducting this study, we hypothesized that patients with a serum 25 (OH) vitamin D level below the normal range would have an increased risk of developing postoperative complications. The purpose of this study was to compare cohorts of osteoporotic hip fracture patients with insufficient and sufficient preoperative 25 (OH) vitamin D levels to assess the impact on postoperative complications after surgical treatment.

## 2. Materials and Methods

### 2.1. Study Design

This study was a retrospective observational cohort study conducted at the Department of Orthopedics and Trauma, Medical University of Graz, Austria. The hospital database was searched for patients who underwent surgical treatment due to a hip fracture in the period between January 2019 and December 2020. Patients were enrolled if they had (i) a fragility fracture from low-energy injury, (ii) located at the proximal femur (pertrochanteric, intertrochanteric, subtrochanteric, proximal femoral shaft, and femoral neck), and (iii) preoperative serum 25-hydroxy vitamin D level was available. Exclusion criteria were (i) secondary hip fracture due to malignancy, (ii) traumatic fracture caused by adequate trauma (e.g., motor vehicle accident), and (iii) non-recent fractures. There were no age restrictions in this study. Eligible patients were followed up from the index date (=date of surgery) to one year after surgical treatment or prior death for any reason.

The present study was approved by the institutional ethics committee of the Medical University of Graz. Due to the retrospective nature of the study, no written or oral consent from the patients was obtained.

### 2.2. Data Collection and Definition

All data were reviewed from electronic medical records, including baseline characteristics such as age, sex, body mass index (BMI), history of malignancy, smoking status, and presence of allergic diseases, as well as the following fracture and treatment-related factors: presurgical American Society of Anesthesiologists (ASA) score, fracture location, surgical procedure, length of hospital stay (in days), and length of operation (in minutes). In addition, vitamin D supplementation prior to surgery was assessed. Preoperative serum 25 (OH) vitamin D level was gathered from the local laboratory charts that were quantified for all patients by chemiluminescence immunoassay (CLIA). An amount of 10 μL of the patient sample is subjected to a pre-treatment step to denature the VDBP, neutralized in assay buffer, and a specific anti-25(OH)D antibody labeled with biotin is added. After incubation, acridinium labeled 25(OH)D is added. Following a further incubation step, the magnetic particles linked to streptavidin are added. After the final incubation step, the complex is captured using a magnet, and a wash step is performed to remove any unbound analyte. Trigger reagents are added, and the resulting light emitted by the acridinium label is inversely proportional to the concentration of 25(OH)D in the original sample. For vitamin D insufficiency, a cut-off of <30 ng/mL was defined as a value below the normal range. Hence, patients were stratified into two groups: a vitamin D-sufficient (normal vitamin D level, ≥30 ng/mL) and a vitamin D-insufficient group (below normal vitamin D level, <30 ng/mL). All postoperative complications were assessed and graded according to the Clavien–Dindo classification system, representing a reliable and compelling tool for quality assessment in surgery [15]. Furthermore, complications were divided into early-onset (those occurring < 30 days after surgery) and late-onset (those developing between 30 days and one year after surgery).

### 2.3. Statistical Analysis

Continuous variables are reported as means with corresponding standard deviations, and categorical variables are presented as frequency distributions and proportions. All variables were compared between the serum 25 (OH) vitamin D level groups (<30 ng/mL, ≥30 ng/mL) using Student’s *t*-test and Mann–Whitney U test for continuous data, as appropriate, and chi^2^-test for categorical data. Additionally, a separate group, including patients with high 25 (OH) vitamin D deficiency (<10 ng/mL), was statistically analyzed. Logistic regression analysis was employed to further analyze the association between preoperative 25 (OH) vitamin D levels (<30 ng/mL, ≥30 ng/mL) and postoperative complications after adjusting for age and sex. In addition, early and late complications were analyzed separately. Odds ratios (ORs) and 95% confidence intervals (CIs) are reported. *p*-values less than 0.05 were considered to be statistically significant. All statistical tests were performed using the software IBM SPSS Statistics version 29.

## 3. Results

In total, 314 patients were evaluated in this study; the mean age was 81.1 years (SD ± 10.2), and 221 (70.4%) were female. The patients’ demographic and clinical characteristics are shown in Table 1. ASA-2 was graded in 31, ASA-3 in 110, and ASA-4 in 82 patients. Osteoporotic fractures were located at the femoral neck (n = 152, 48.4%), femoral shaft (n = 2, 0.6%), peritrochanteric area (n = 147, 46.8%), and subtrochanteric area (n = 13, 4.1%). The most common operative procedures were intramedullary nails (n = 168, 53.5%), followed by hemiprosthesis (n = 109, 34.7%). In 18.2% (n = 57) of cases a cement augmentation was performed. The mean duration of surgery was 54.9 min (SD ± 32.0), and overall hospitalization was 8.9 days (SD ± 5.7).

### 3.1. Preoperative 25 (OH) Vitamin D Levels

The mean serum 25 (OH) vitamin D level was 22.6 ng/mL (SD ± 13.2) and was found to be below the normal range of <30 ng/mL in 222 patients (70.7%). A total of 92 patients (29.3%) had a preoperative 25 (OH) vitamin D level of >30 ng/mL. Vitamin D levels were <30 ng/mL in 151 female and 71 male patients, with no significant difference between the groups (*p* = 0.154). Furthermore, there was no significant difference between 25 (OH) vitamin D-sufficient and -insufficient groups in terms of age, history of malignancy, smoking status, presence of allergic diseases, surgical procedure, duration of hospitalization and surgery. A significant difference was detected in the BMI (*p* = 0.007), with a higher mean BMI in patients with preoperative 25 (OH) vitamin D levels <30 ng/mL and in the ASA score (*p* = 0.01). While most patients with low vitamin D levels (<30 ng/mL) scored ASA 3 (68.3%), patients with normal vitamin D levels (≥30 ng/mL) were mostly categorized as ASA 4 (55.4%). In addition, 15.2% of patients received vitamin D (cholecalciferol) and 3.8% bisphosphonates prior to surgery, with significant differences between the groups (*p* = 0.006, *p* = 0.001). Calcium was supplemented in 9.5% and 16.3% of vitamin D-deficient (<30 ng/mL) and -sufficient patients (≥30 ng/mL), respectively (*p* = 0.08).

### 3.2. Postoperative Complications

Overall, postoperative complications were observed in 116 patients (36.9%). In terms of short-term complications, 95 patients (30.2%) developed adverse events within 30 days of surgery. Of these, 16.8% were rated as Clavien I, 61.1% as Clavien II, 6.3% as Clavien IIIb, 2.1% as Clavien IVa, and 13.7% as Clavien V (Table 2). The most common complications were postoperative anemia < 7 ng/mL requiring postsurgical blood transfusion (n = 42 patients), postoperative urinary tract infection (n = 9), early prosthetic joint infection (n = 6), and pneumonia (n = 5) (Table 3). Thirteen patients (4.1% of included patients) died after surgery during hospitalization. The mean duration of hospitalization for 25 (OH) vitamin D-deficient patients with early postoperative complications (11.0 ± 8.1 days) was different but not statistically significant (*p* = 0.729) compared to those without early adverse events (8.2 ± 4.9 days).

Late postoperative complications occurring between 30 days and 1 year of follow-up were found in 21 patients (6.7%). With regard to the Clavien–Dindo classification system, late complications were commonly graded as Clavien I (57.1%), followed by Clavien 3b (28.6%), Clavien 2 (9.5%), and Clavien 4a (4.8%) (Table 1). The most common complications were related to surgery or the implant, such as heterotopic ossification, thrombosis, implant fracture, implant irritation or migration, or loosening of the prosthesis (Table 4).

Logistic regression analysis revealed a significantly higher risk of early postoperative complications with 76 patients (34.2%) in the insufficient group and 19 patients (20.6%) in the sufficient group (OR 2.06, 95% CI 1.14–3.73, *p* = 0.016). In 15 of 21 patients with late complications preoperative 25 (OH) vitamin D levels were under 30 ng/mL, but no significant correlation was found (OR 1.08, 95% CI 0.40–2.95, *p* = 0.879).

If we focus on patients with vitamin D levels <10 ng/mL, the complication rate increases further (*p*-value for early complication is 0.012 and 0.025 for overall complication rate).

## 4. Discussion

The data of this study show a possible correlation between insufficient preoperative vitamin D levels and a significant risk of early postoperative complications. Considering the high mean age of osteoporotic hip fracture patients, a low preoperative vitamin D level of <30 ng/mL was prevalent in 70.7% of cases. Similarly, the low mean 25 (OH) vitamin D level in our study cohort was comparable to the widespread prevalence of vitamin D deficiency reported in orthopedic cohorts [16,17,18] and in osteoporotic patients [19].

In the present study, osteoporotic hip fracture patients with vitamin D status below the normal range (<30 ng/mL) had a significantly higher rate of early postoperative complications than patients with normal vitamin D levels. Consistently, the tendency for decreasing vitamin D levels to correlate with increasing complication rates has been observed in other studies. In a systematic review by Iglar et al., 26 of 31 included studies reported at least one statistically significant medical complication following diverse surgical procedures (i.e., gastric surgery, cardiac surgery, pulmonary surgery, kidney transplant, hip/knee/shoulder endoprothetic surgery) in patients with low vitamin D levels [20]. In addition, Fakler et al. found 20% of hip fracture patients with in-hospital postoperative complications with vitamin D as an independent risk factor (OR 0.89, 95% CI 0.81–0.97; *p* = 0.010) [10].

The present study shows the diversity and severity of adverse outcomes in patients undergoing osteoporotic hip fracture surgery, such as cardiac, infectious, pulmonary, or surgery-related problems that are comparable to short-term complications of previous studies with different patient cohorts [11,12,21]. In our analysis, the most common complication in the short term was postsurgical anemia requiring blood transfusions due to hemoglobin levels <7 mg/dL. Postoperative anemia may be caused by worsening of preoperative anemia, peri-operative blood loss, and postoperative reduced erythropoiesis due to surgery-related inflammation. In addition, inflammation processes lead to diminished gastrointestinal iron uptake and a diminished erythroid response to erythropoietin, respectively, which could further delay the recovery of hemoglobin postoperatively [22]. Moreover, several observational studies have shown the impact of vitamin D on anemia. In the cross-sectional study by Ernst et al., an independent inverse association between circulating 25 OH vitamin D level and anemia risk after cardiac surgery was found [23]. As vitamin D is known to be a modulator of systemic cytokine production, it may reduce the inflammatory milieu that causes anemia of chronic diseases. Sim et al. demonstrated that 25 OH-deficient patients had a lower mean Hb (*p* = 0.12) and a higher prevalence of erythrocyte-simulating agent use (*p* < 0.05) [24]. Furthermore, serum 1,25 (OH) vitamin D stimulates erythropoiesis, affects bone marrow mesenchymal stem cells (BM-MSCs), and thus influences immunomodulation and hematopoiesis [25,26]. In addition, these cells modulate immune reactions in case of infection and injury by producing various factors, such as cytokines, extracellular vesicles, and growth factors, which are crucial in the treatment of osteoporosis [26]. As such, it is tempting to conclude that vitamin D supplementation would be of two-fold benefit for hip fracture patients with respect to postoperative anemia requiring blood transfusions and osteoporosis.

Furthermore, in this study, a high number of postoperative complications were linked to various infectious diseases, including periprosthetic joint infection, pneumonia, and postoperative urinary tract infection. Similar results were found in previous studies. Maier et al. reported a significantly higher frequency of vitamin D deficiency in patients with periprosthetic joint infection following primary arthroplasty [16]. In a retrospective study, Lim et al. found a significantly higher risk of pneumonia in hip fracture patients with vitamin D levels < 20 ng/mL (OR 3.82, 95% CI 1.29–11.34) [11]. Additionally, Zhou et al. reported an association between vitamin D deficiency and an increased risk of community-acquired pneumonia [27]. Notably, the immunomodulatory role of vitamin D and 1,25 OH has gained much interest in recent literature. In vivo studies have demonstrated the biological effect of vitamin D on both the innate and adaptive immune system, suggesting that vitamin D deficiency may contribute to immune response dysregulation [28]. Vitamin D stimulates the production of pattern recognition receptors (PRRs), antimicrobial peptides, and cytokines in the cells, while preventing the maturation and activation of dendritic cells as well as the differentiation of monocytes into macrophages. In addition, 1,25 OH attenuates the production of IL-2, IFN γ, and T-helper type 1 cytokines. It enhances the synthesis of Th2 anti-inflammatory cytokines and decreases the production of Th9 (IL-9) and TH22 (IL-22) pro-inflammatory cytokines [29]. Thus, patients with low vitamin D levels may have an increased susceptibility to bacterial and viral postoperative infections.

Another significant finding represents the high rate of in-hospital mortality, which is above the rate reported in many other studies. Groff et al. described an overall in-hospital mortality rate of 3.0% in patients undergoing surgical treatment due to an acute hip fracture [30]. Additionally, a case–control study conducted by Chiang et al. noted that 2.0% of patients (mean age 86.5 years) died in the hospital after hip fracture surgery [31]. In contrast, a large Brazilian retrospective study on osteoporotic hip fracture patients revealed an in-hospital mortality of 18.4%, with the highest incidence in patients with some type of infection (34%) and a significantly higher risk of mortality (PR = 1.54; 95% CI 1.03–2.29) in patients with hemoglobin levels ≤ 10 g/dL [32]. While various factors may contribute to patients’ survival after surgical procedures, it is worth mentioning that 12 of 13 patients in the present study had a vitamin D level below the normal range (<30 ng/mL). A large body of evidence has demonstrated that higher postoperative mortality rates among hip fracture patients are linked to lower levels of vitamin D at 30 days [33], 1 year [34,35,36,37], and 2 years [36] of follow-up. The pooled data of a recent meta-analysis on femur fracture patients revealed that vitamin D insufficiency and severe deficiency are associated with increased mortality in patients with hip fractures. Notably, there was no statistically significant increase in mortality after adjusting for confounding factors. A further subgroup analysis pointed out that vitamin D insufficiency is associated with significantly higher mortality rates at 1- and 2-year follow-ups, whereas severe deficiency was significantly associated with mortality in the short term [38]. In this respect, it is important to question whether vitamin D solely acts as a surrogate for populations’ general health status. Considering the present ASA score results, we found that patients with normal levels of vitamin D had significantly higher ASA scores than patients with vitamin D insufficiency but did not experience higher mortality rates as anticipated. Therefore, we postulate that vitamin D may play a major role in complications and survival in the short term.

However, vitamin D was not confirmed as an independent predictor for late postoperative complications. Even though most adverse events up to 1 year are related to surgery, or the implant itself, vitamin D status surprisingly did not have a substantial impact. The essential role of vitamin D in bone mineralization and maintenance of bone quality through regulating calcium and skeletal homeostasis is well known. Furthermore, bone mineralization is a crucial part of callus formation and bone remodeling in the process of fracture healing [39]. A recent systematic review on the effect of vitamin D supplementation on bone healing in fracture patients found that only three of eight studies reported a positive effect of vitamin D supplementation on clinical unions among fracture patients. Consistent with our findings, one of these studies showed differences in early but not in late orthopedic complications [40]. In addition, Kenanidis et al. showed that patients with a lower vitamin D level had a higher risk of postoperative joint stiffness and periprosthetic joint infection [41]. Nevertheless, there is little evidence concerning the impact of vitamin D on fracture healing and complications, especially in patients with osteoporosis. It would be of further research interest to investigate whether vitamin D in combination with calcium or osteoporosis medication is effective in preventing long-term complications.

Due to the high likelihood of the population aged 60 years and above experiencing an osteoporotic hip fracture with a substantial risk for postoperative complications in their further lifetime course, targeted interventions in vulnerable populations are needed. The accurate timing of dosage is of clinical importance and has been investigated in previous studies. While some researchers showed a rapid rise in 25 (OH) vitamin D levels following a single high-dose bolus of 300,000 IU [42,43], the systematic review by Iglar et al. stated that only minimal benefits for long-term outcomes could be gained by supplementation at the time of surgery [20]. Additionally, the beneficial effect on several body systems prior to surgery that affect the medical outcome would otherwise be missed (i.e., low hemoglobin level). In this context, it is worth noting that many complications in the postoperative course may be related to comorbidities that were present before surgery [44] and could be associated with a low vitamin D status as well. Since osteoporotic hip fracture surgery may not be scheduled weeks ahead and a daily supplementation for at least 3 months prior to surgery is needed to increase 25 (OH) vitamin D serum at the long-term follow-up [14], treatment strategies should be included in public health interventions. Future regular health checkups provided by the government may take up a screening test for vitamin D deficiencies and subsequent supplementations if needed. A simple, cheap, and safe treatment could improve the postsurgical course in terms of complications and survival in patients with osteoporotic hip fractures. Nevertheless, more research is warranted to develop effective prevention and treatment strategies. Another reason for a high-dosage vitamin D refilling protocol is to start an antiresorptive or osteoanabolic therapy as early as possible after surgery to prevent further fractures. Therefore, at least low default levels of vitamin D are mandatory.

There are some limitations to the present study. First, due to the retrospective nature of the design, data accuracy may be limited. Acquired data were not primarily registered for research purposes, and clinical notes were taken from different physicians, which invariably led to inter-observer bias. Second, the follow-up in the long-term course was restricted as patients may have consulted other hospitals due to possible detrimental surgery-related problems. Hence, we expect a small quantity of under-reporting in terms of late complications. In order to limit this bias, we thoroughly reviewed the medical charts of all trauma departments of the same hospital operator. Another limitation concerns the comparison with previous studies due to heterogeneity in cut-off points for proposing vitamin D insufficiency and deficiency. Study results should be interpreted and generalized with caution, as the definition of low vitamin D levels differs widely in the existing evidence. Many experts have defined vitamin D deficiency as a 25 (OH) vitamin D level of less than 20 ng/mL [45,46], but no consensus on optimal levels exists as a threshold may not be generalizable for all populations and health conditions. In this study, we defined a cut-off point of 30 ng/mL due to the evidence of osteoporosis in the study population that is in a causal relationship to hypovitaminosis [47]. As stated by the Mayo Clinic, a mild-to-modest 25 (OH) vitamin D deficiency can be associated with osteoporosis [48]. Additionally, a high parathyroid hormone level is associated with an increased risk for osteoporosis and osteoporotic fractures [49]. 25 (OH) vitamin levels may suppress parathyroid secretion until they reach values above 30 ng/mL [50]. Moreover, the American Association of Clinical Endocrinologists [51] has defined vitamin D deficiency as a 25 (OH) level below 30 ng/mL, and the American Geriatrics Society has proposed achieving a minimum concentration of 30 ng/mL in frail elderly individuals who are at higher risk of falls, injuries, and fractures [52]. Moreover, preoperative vitamin D levels extracted from laboratory charts had a defined lower limit of <4 ng/mL that is inherently linked to the so-called floor effect, and an overestimation of mean 25 (OH) vitamin D level could not be excluded. The major strength of this study is the relatively high number of patients and the focus on injuries that are highly connected with osteoporosis.

## 5. Conclusions

In osteoporotic hip fracture patients, preoperative 25 (OH) vitamin D serum level might be an independent predictor of early postoperative complications. However, no association between preoperative 25 (OH) vitamin D level and long-term complications could be found. Public health interventions should strive for a sufficient serum level to reduce grievous short-term outcomes following surgery. Additionally, large-scale prospective studies are needed to determine potential risk factors for long-term outcomes in osteoporotic hip fracture patients and to establish appropriate treatment strategies.

## Figures and Tables

**Table 1:** Patient´s baseline characteristics stratified according to serum 25 (OH) vitamin D level.

	Total	Vitamin D High-Insufficient Group (<10 ng/mL)	Vitamin D-Insufficient Group (<30 ng/mL)	Vitamin D- Sufficient Group (≥30 ng/mL)	*p*-Value <30 ng/mL vs. ≥30 ng/mL
no. of patients	314	73 (23.2%)	222 (70.7%)	92 (29.3%)	
age (years)	81.1 (±10.2)	81.3 (±10.1)	81.43 (±9.8)	80.13 (±11.1)	0.31
gender					
male	93 (29.6%)	22 (30.1%)	71 (32.0%)	22 (23.9%)	
female	221 (70.4%)	51 (69.9%)	151(68.0%)	70 (76.1%)	0.15
BMI	23.9 (±4.1)	24.0 (±4.0)	24.3 (±4.1)	23.0 (±4.0)	0.007 *
BMI > 30	21 (6.7%)	6 (8.2%)	17 (7.7%)	4 (4.3%)	0.28
history of malignancy	48 (15.3%)	11 (15.1%)	34 (70.8%)	14 (29.2%)	0.98
current smoker	43 (13.7%)	8 (11.0%)	30 (69.8%)	13 (30.2%)	0.89
allergic disease	78 (24.8%)	16 (21.9%)	57(73.1%)	21 (26.9%)	0.60
ASA scores					
2	31 (13.9%)	4 (5.5%)	17 (16.3%)	14 (11.8%)	
3	110 (49.3%)	22 (30.1%)	71 (68.3%)	39 (32.8%)	
4	82 (36.8%)	28 (38.4%)	16 (15.4%)	66 (55.4%)	0.01 *
fracture location					
femoral neck	152 (48.4%)	40 (54.8%)	111 (50%)	41 (44.5%)	0.38
proximal femoral shaft	2 (0.6%)	0 (0.0%)	0 (0%)	2 (2.2%)	0.03 *
peritrochanteric	147 (46.8%)	30 (41.1%)	100 (45.0%)	47 (51.1%)	0.33
subtrochanteric	13 (4.1%)	3 (4.1%)	11(5%)	2 (2.2%)	0.26
surgical procedure					
intramedullary nail	168 (53.3%)	36 (49.3%)	117 (52.7%)	50 (54.4%)	0.85
hemiprosthesis	109 (34.6%)	32 (43.9%)	80 (36.0%)	29 (31.5%)	0.44
total endoprosthesis	15 (4.8%)	2 (2.7%)	11 (5.0%)	4 (4.3%)	0.82
femoral neck system	15 (4.8%)	2 (2.7%)	9 (4.1%)	6 (6.5%)	0.35
screw fixation	8 (2.5%)	1 (1.4%)	5 (2.2%)	3 (3.3%)	0.82
cement augmentation	57 (18.2%)	15 (20.5%)	43 (57.4%)	14 (24.6%)	0.39
duration of surgery (min)	54.9 (±32.0)	55.8 (±28.5)	56.3 (±34.5)	51.6 (±25.1)	0.18
length of hospitalization (days)	8.9 (±5.7)	9.3 (±5.4)	9.1 (±6.2)	8.7 (±4.5)	0.52
preoperative vitamin D supplementation ^a^	48 (15.2%)	5 (6.8%)	26 (11.7%)	22 (23.9%)	0.006 *
preoperative calcium supplementation	36 (11.5%)	3 (4.1%)	21 (9.5%)	15 (16.3%)	0.08
preoperative bisphosphonate intake	12 (3.8%)	0 (0.0%)	3 (1.3%)	9 (9.8%)	0.001 *
preoperative 25 (OH) vitamin D level (ng/mL)	22.6 (±13.2)	6.1 (±2.0)	16.0 (±8.5)	38.6 (±7.5)	<0.001 *
preoperative calcium serum level (mg/dL)	2.3 (0.2%)	2.3 (±0.1)	2.3 (±0.2)	2.3 (±0.1)	0.14

Data are presented as number (%) or mean (±standard deviation). BMI, body mass index; ASA, American Society of Anesthesiologists; ^a^ patients supplemented cholecalciferol drops prior to surgery; * variables with *p*-value < 0.05.

**Table 2:** Number of patients with postoperative early and late complications, including Clavien–Dindo classification.

	Total	Vitamin D High-Insufficient Group (<10 ng/mL)	Vitamin D-Insufficient Group (<30 ng/mL)	Vitamin D-Sufficient Group (≥30 ng/mL)	OR (95% CI) <30 ng/mL vs. ≥30 ng/mL	*p*-Value <30 ng/mL vs. ≥30 ng/mL
postoperative complications	116	32 (43.8%) # *p* = 0.025	91 (41.0%)	25 (27.2%)		
early complications	95	28 (38.4%) # *p* = 0.012	76 (34.2%)	19 (20.6%)	2.06 (1.14–3.73)	0.016 *
Clavien–Dindo Scale						
1	16 (16.8%)	4 (14.3%)	14 (18.4%)	2 (10.5%)		
2	58 (61.5%)	17 (60.7%)	46 (60.5%)	12 (63.1%)		
3b	6 (6.3%)	0 (0.0%)	3 (4.0%)	3 (15.8%)		
4a	2 (2.1%)	0 (0.0%)	1 (1.3%)	1 (5.3%)		
5	13 (13.7%)	7 (25.0%)	12 (15.8%)	1 (5.3%)		
late complications	21	4 (3.4%)	15 (6.8%)	6 (6.5%)	1.08 (0.40–2.95)	0.879
Clavien–Dindo Scale						
1	12 (57.1%)	2 (50.0%)	7 (46.7%)	5 (83.3%)		
2	2 (9.5%)	1 (25.0%)	2 (13.3%)	0 (0.0%)		
3b	6 (28.6%)	1 (25.0%)	5 (33.3%)	1 (16.7%)		
5	1 (4.8%)	0 (0.0%)	1 (6.7%)	0 (0.0%)		

Data are presented as the number (%) of patients experiencing postoperative complications. Logistic regression analysis: OR, odds ratio; CI, confidence interval. * Variables with *p*-value < 0.05; # *p*-value < 10 ng/mL vs. ≥30 ng/mL.

**Table 3:** Early postoperative complications according to serum 25 (OH) vitamin D level group.

	Total	Vitamin D-Insufficient Group (<30 ng/mL)	Vitamin D-Sufficient Group (≥30 ng/mL)
acute on chronic kidney failure	1 (0.95%)	0 (0.00%)	1 (5.26%)
increased inflammation parameters	3 (2.86%)	2 (2.33%)	1 (5.26%)
urinary tract infection	8 (7.62%)	7 (8.14%)	1 (5.26%)
early periprosthetic joint infection	6 (5.71%)	3 (3.49%)	3 (15.79%)
pneumonia	4 (3.81%)	4 (4.65%)	0 (0.00%)
postoperative anemia ^a^	42 (40.00%)	33 (38.37%)	9 (47.37%)
intracerebral hemorrhage	1 (0.95%)	1 (1.16%)	0 (0.00%)
cardiac decompensation	2 (1.90%)	2 (2.33%)	0 (0.00%)
myocardial infarction	1 (0.95%)	1 (1.16%)	0 (0.00%)
pulmonary embolism	1 (0.95%)	0 (0.00%)	1 (5.26%)
syncope	1 (0.95%)	1 (1.16%)	0 (0.00%)
thrombosis	3 (2.86%)	0 (0%)	0 (0.00%)
decubitis	1 (0.95%)	1 (1.16%)	0 (0.00%)
dermatitis	3 (2.86%)	0 (0%)	0 (0.00%)
edema	1 (0.95%)	1 (1.16%)	0 (0.00%)
seroma	3 (2.86%)	2 (2.33%)	1 (5.26%)
hematoma	4 (3.81%)	4 (4.65%)	0 (0.00%)
wound healing disorder	3 (2.86%)	0 (0%)	0 (0.00%)
herpes	1 (0.95%)	1 (1.16%)	0 (0.00%)
increased creatinine	1 (0.95%)	1 (1.16%)	0 (0.00%)
implant failure	1 (0.95%)	1 (1.16%)	0 (0.00%)
shortening of the femoral shaft	1 (0.95%)	0 (0.00%)	1 (5.26%)
exitus letalis	13 (12.38%)	12 (13.95%)	1 (5.26%)

Data are presented as the number (%) of early postoperative complications; ^a^ postoperative anemia < 7 g/dL with transfusion need.

**Table 4:** Late postoperative complications according to serum 25 (OH) vitamin D level group.

	Total	Vitamin D-Insufficient Group (<30 ng/mL)	Vitamin D-Sufficient Group (≥30 ng/mL)
heterotopic ossification	4 (19.05%)	3 (20.00%)	1 (16.67%)
thrombosis	2 (9.52%)	2 (13.33%)	0 (0.00%)
bursitis	1 (4.76%)	0 (0.00%)	1 (16.67%)
hematoma	2 (9.52%)	1 (6.67%)	1 (16.67%)
seroma	1 (4.76%)	0 (0.00%)	1 (16.67%)
implant fracture	2 (9.52%)	2 (13.33%)	0 (0.00%)
implant irritation	1 (4.76%)	1 (6.67%)	0 (0.00%)
implant migration	3 (14.29%)	2 (13.33%)	1 (16.67%)
implant cut-out	1 (4.76%)	0 (0.00%)	1 (16.67%)
implant loosening	1 (4.76%)	1 (6.67%)	0 (0.00%)
secondary fracture dislocation	1 (4.76%)	1 (6.67%)	0 (0.00%)
impaired wound healing	1 (4.76%)	1 (6.67%)	0 (0.00%)
hemorrhagic shock	1 (4.76%)	1 (6.67%)	0 (0.00%)

Data are presented as the number (%) of late postoperative outcomes.

## Data Availability

The data presented in this study are available on request form the corresponding author due to privacy and legal reasons.
